# Supplementary figures and images for: Coordinated regulation of Cdc42ep1, actin, and septin filaments during neural crest cell migration
Source: Front Cell Dev Biol. 2023 Feb 27;11:1106595. doi: 10.3389/fcell.2023.1106595 (PMC10009165; doi:10.3389/fcell.2023.1106595)

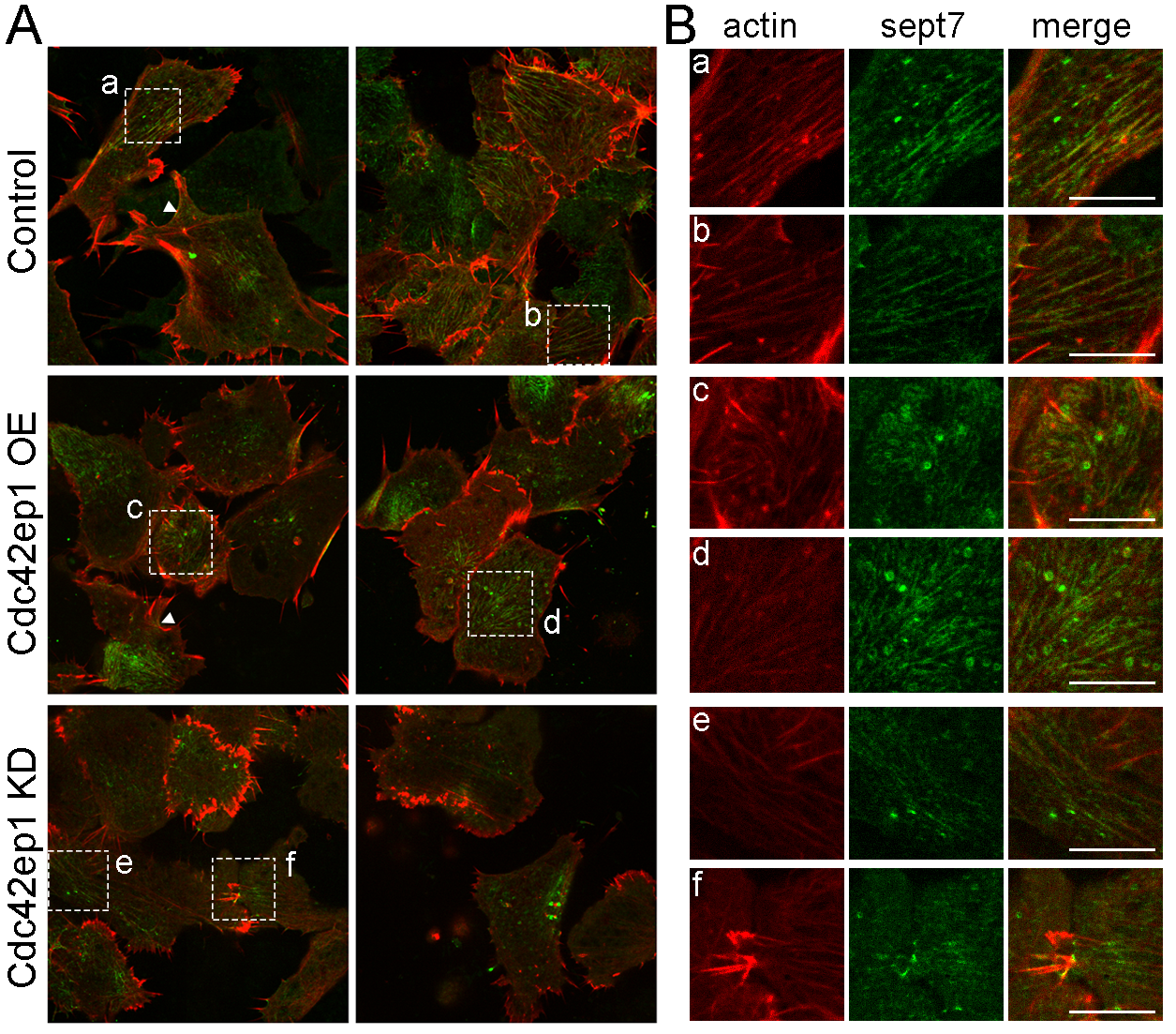

Supplement: Supplementary file 4 [file Image3.TIF]

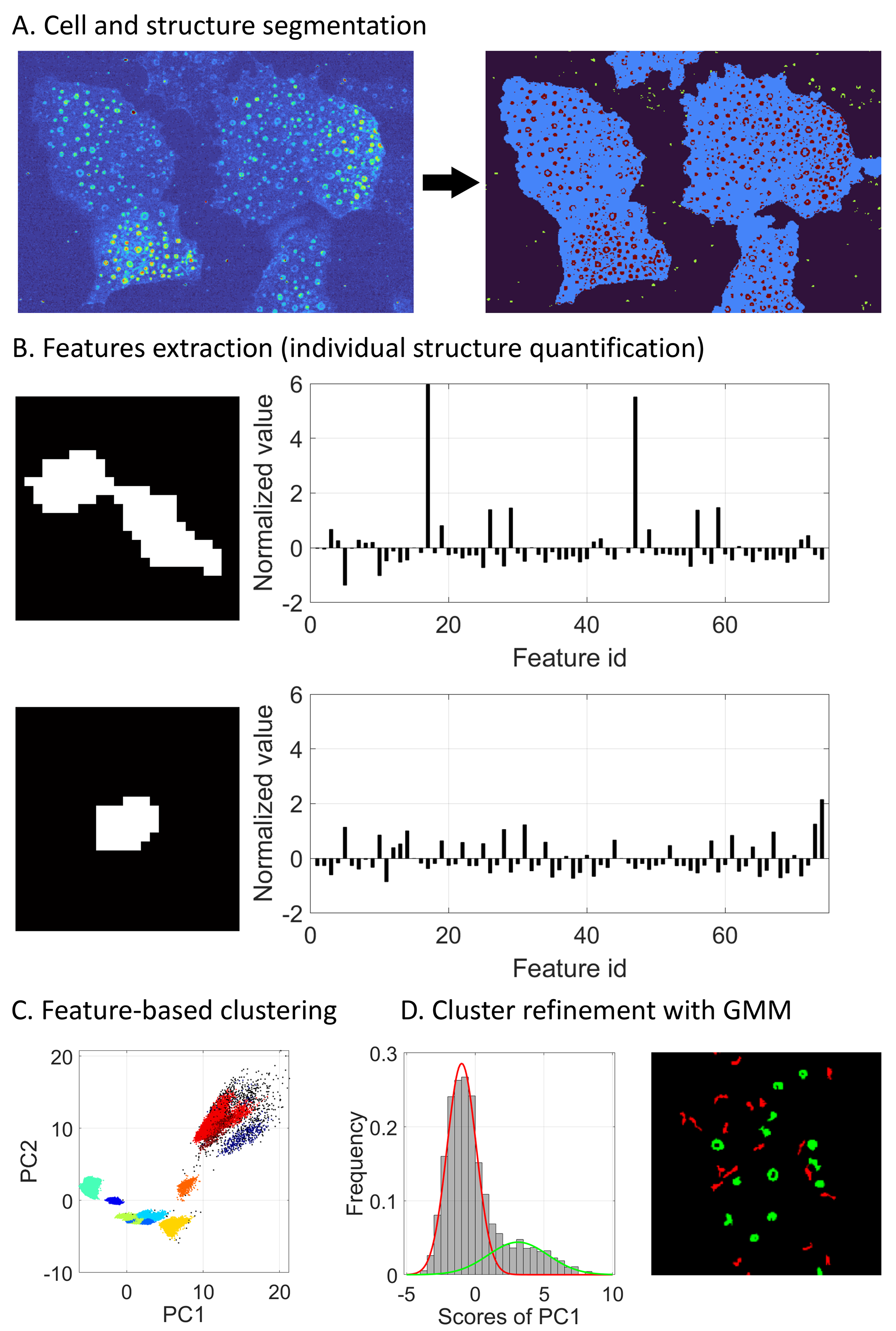

Supplement: Supplementary file 5 [file Image4.TIF]

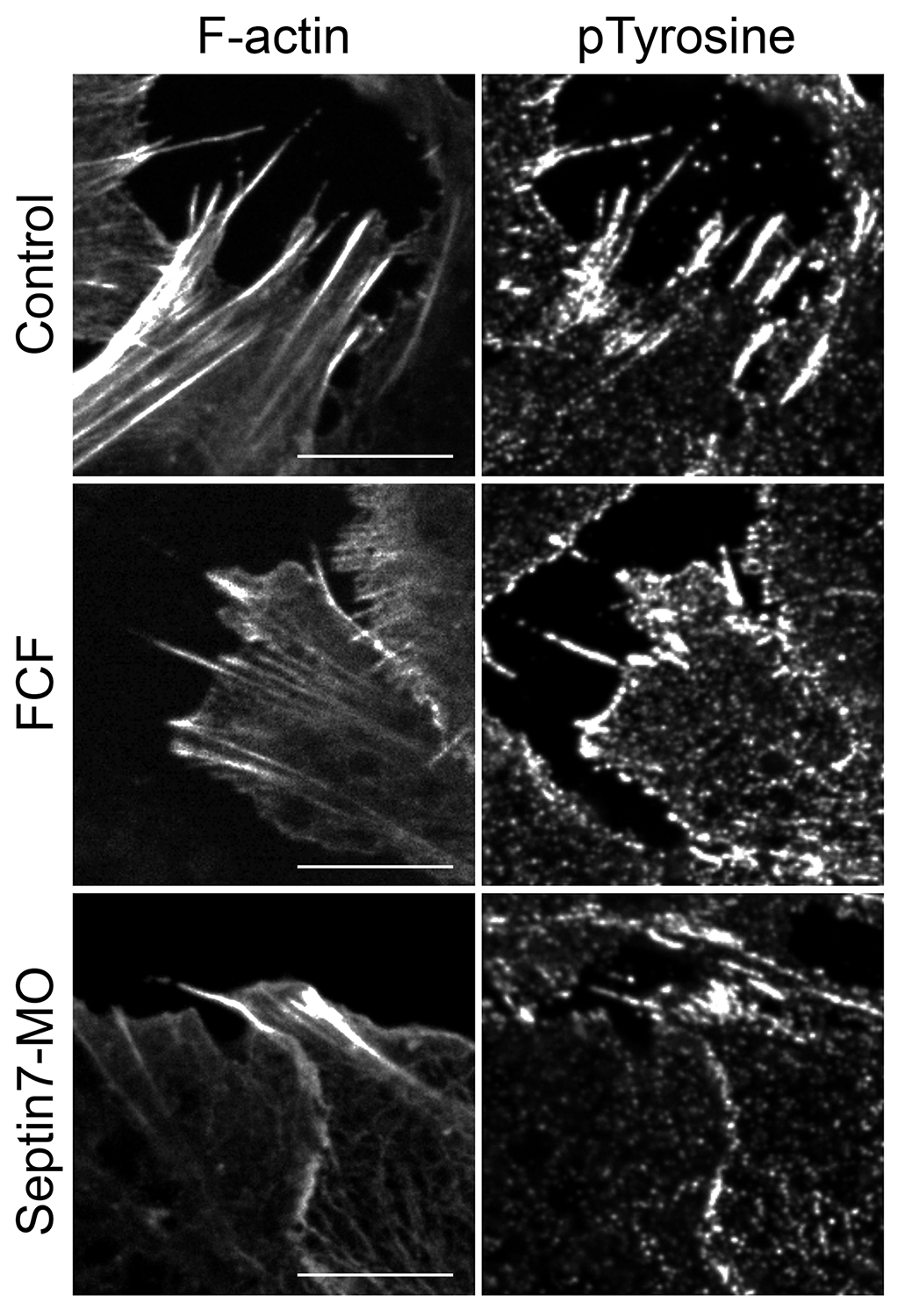

Supplement: Supplementary file 6 [file Image2.TIF]

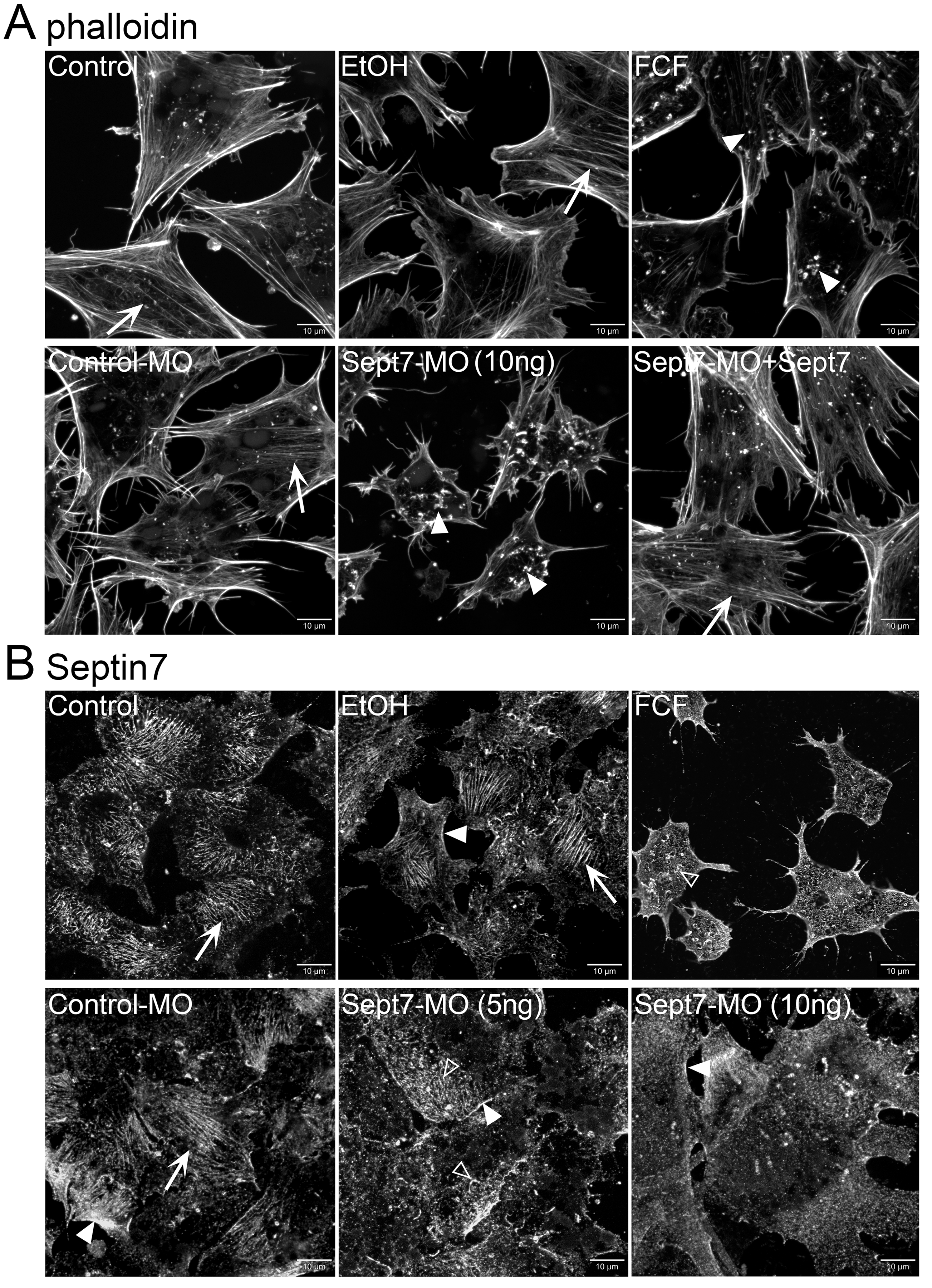

Supplement: Supplementary file 7 [file Image1.TIF]

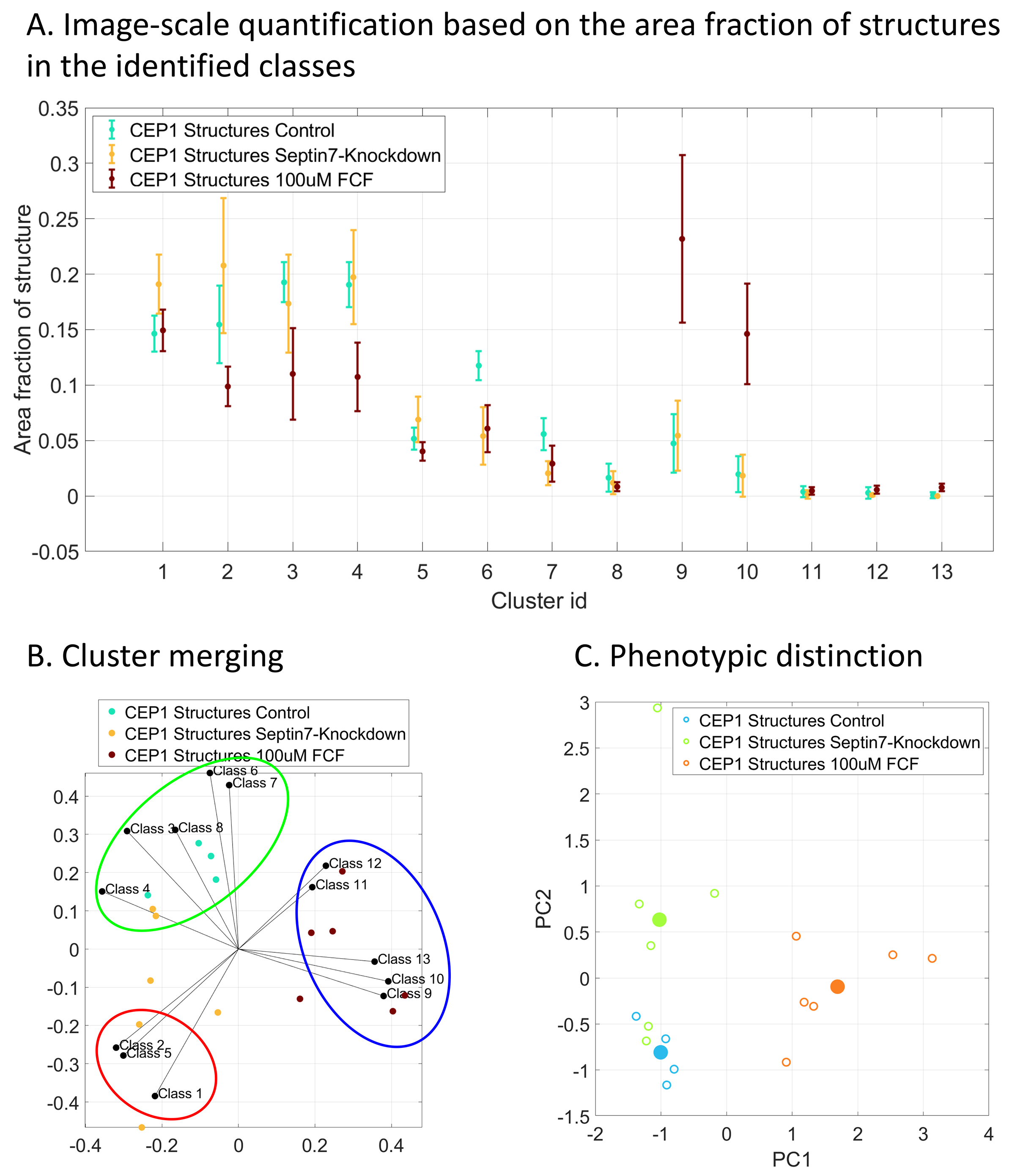

Supplement: Supplementary file 10 [file Image5.TIF]
